# Practical Notes and Formulæ

**Published:** 1879-08-20

**Authors:** 


### PRACTICAL NOTES AND FORMULÆ.

**Skin Diseases.**—In Squire's Pharmacopœia of the British Hospital we find the following:

CHRONIC ECZEMA.

A carbolic acid lotion, gr. x, to water ℥ j, is used to allay irritation. As a mild stimulant, a pigment of aloes.

℞ Aloes extracti barbad.............................. ʒ iij.

Glycerini.............................................. ʒ vj.

Dissolve.

An ointment of birch oil is used cautiously in dry eczema.

℞ Olei betulæ albæ.................................. ʒ ij.

Ceræ flavæ.......................................... ʒ ij. Melt.

In subacute and chronic forms, the oleate of zinc is soothing.

℞ Zinci oleatis........................................ ʒ ij.

Adipis.................................................. ℥ j.

CHLOASMA LENTIGO.

For these troublesome discolorations of the skin an ointment of alizarin is recommended.

℞ Alizarini factitii.................................. ʒ j.

Adipis.................................................. ℥ j. M

Or a sulphurous acid lotion.

℞ Acidi sulphurosi.................................. ʒ ij.

Aquam ad............................................ ℥ j. M

The rapid evaporation of the acid from the lotion should be retarded by applying the lotion on lint or rag covered with oil silk.

As a bleaching agent in lentigo:

℞ Sodæ chloratæ liquoris.......................... ʒ ss.

Aquæ.................................................. ℥ j. M

Chrysophanic acid ointment is also useful in these affections.

PSORIASIS.

Chrysophanic acid is a leading remedy.

℞ Acidi chrysophanici.............................. ʒ ij.

Adipis.................................................. ℥ j.

Heat together in a water bath for half an hour; when set, mix with pestle and mortar.

The erythema which arises after some times only a few applications of this ointment should be watched for, and on its first appearance the use of the ointment should be promptly discontinued. It should never be applied in the vicinity of the eyelids, as they are apt to be rendered œdematous.

**Palatable Castor Oil.**—Rub two drops of oil of cinnamon with an ounce of glycerine, and add one ounce of castor oil. Children will take it as a luxury and ask for more.—*Courier of Medicine.*

**Misturæ.**—The Pharmacopœia of the Hospital of the University of Pennsylvania gives :

*Mistura Potassii Cyanidi.*

℞ Potassii cyanidi........................................... gr. j.
Syr. acidi citrici.......................................
Aquæ...........................................aa f ℥ j.

Dose, one teaspoonful.

*Mistura Sodii Composita.*

℞ Sodii bicarbonatis........................................ gr. xx.

Acidi carbolici............................................ gtt ij.

Acaciæ..................................................

Sacchari..........................................aa q. s.

Spts. lavendulæ com...................................... f ʒ ij.

Aquæ.................................................. q. s. ad f ℥ j. M

Dose, a teaspoonful two hours after meals.

*Mistura Olei Morrhuæ.*

℞ Olei morrhuæ............................................ f ℥ j.

Tinc. iodinii comp...................................... m viij. M

Dose, one to four teaspoonfuls.

*Mistura Olei Morrhuæ et Ætheris.*

℞ Mist. ol. morrhuæ...................................... f ℥ j.

Ætheris.................................................. m xvj. M

Dose, one to four teaspoonfuls.

*Mistura Olei Morrhuæ et Calcis Lacto-Phosphatis.*

℞ Olei morrhuæ..........................................

Mucil acaciæ..........................................aa f ʒ ij.

Fiat emulsio et adde :

Syrupi calcis lacto-phosphatis.......................... f ℥ ss

*Mistura Hyoscyami et Morphiæ.*

℞ Morphia acetatis........................................ gr. ¼.

Tinc. hyoscyami.......................................... f ʒ iss.

Syr. tolutana............................................ f ʒ ijss.

Aqua..................................................... f ℥ ss.

Dose, two teaspoonfuls.

*Mistura Ammonii Chloridi.*

℞ Ammonii chloridi....................................... xx.

Aceti scilla.............................................. f ʒ j.

Mist. Glycyrrhiza comp.................................. f ʒ vij.

Dose, two to four teaspoonfuls.

*Mistura Cinchoniæ Acida.*

℞ Cinchonia sulphatis.................................... gr. iv.
Acidi nitromuriatici dil................................. m xij.
Aqua cinnam.......................................... q. s. ad. f ℥ j.

Faciat solutio. Dose, a tablespoonful in water, before meals.

—*Hospital Gazette.*

**Hog Cholera.**—Dr. A. W. Burrows, of Ind., writes :

The following are the ingredients used in Dr. Jos. Haas' hog cholera remedy, which is used extensively in the West for arresting disease so prevalent among swine :

| | | |
|---|---|---|
| Ashes | 20 | parts. |
| Lime | 20 | " |
| Salt | 1 | " |
| Soda | 2 | " |
| Baberry | 2 | " |
| Capsicum | 2 | " |
| Arsenic | 1 | " |
| Calomel | 1 | " |
| Spanish brown | 6 | " |
| Flour | 3 | " |
| Tonca bean | 1 | " |

The remedy has proven more efficient than anything else ever used, and I place it at your disposal, believing that it will be of interest to many of your readers. It should be given as follows :

One even tablespoonful twice a day for each hog when sick. When used as a preventive, one tablespoonful for each three hogs once or twice a week.

**Malarial Fever of the South.**—L. A. Rutherford, M.D., of Macon, Ga., writes :

There is doubtless an endemic modification of yellow fever pervading yearly certain rural districts of the South, requiring only the crowding and filth of large cities to entail upon it the fatally epidemic character of its city cousin. In this pathological condition we have, superadded to the ordinary malarial poisoning, cholæmia, or the accumulation in the circulating fluid of cholesteriane and other excrementitious properties of the bile. If this retrograde metamorphosis of the vital fluid be not speedily arrested by suitable hæmatics, we shall invariably have necræmia and speedy dissolution.

The treatment should point to the removal of all offending material by prompt and stimulating emetics, which will fulfil the twofold purpose of restoring vital function, and removing in part the causa morbi. The early, free and persistent use of quinine, or dextro-quinine, beef tea, counter-irritation and hot fomentations, will be the clear line of practice. I have used the new antiperiodic, dextro-quinine, with much success in the treatment of such cases.

**Potato and Diphtheria.**—Dr. W. W. Carpenter, of California, writes :

The May issue of the RECORD contains a selected article from the Medical Reporter, written by Dr. M. C. Keith, of Nebraska, entittled "Potato Eating and Diphtheria." My object is not to refute the Doctor's theory further than to suggest that in so much as potatoes have been the universal food of childhood from time immemorial, it is somewhat strange that so many of us have survived the ordeal.

The Doctor rests his position on a personal experience of seventeen years, and an experience of twelve years in his father's practice, Dr. Alvan Keith, of Augusta, Me. The father's experience was all previous to 1861. Now, when we reflect that diphtheria first presented itself to this generation of physicians in 1856; that the first point of invasion on this continent was in this state and this town (Petaluma); that it raged here two years before it reached the Atlantic coast; that not a case occurred in Maine until 1859, will Dr. Keith explain how his father acquired twelve years' experience in its treatment prior to 1861?

**Dressing Head Wounds.**—Dr. Smith, in Nashville Medical Journal, says:

Our favorite plan, especially in head wounds, is to draw the lacerated parts together with silver wire sutures, and keep the parts wet with paregoric and thin simple syrup equal parts, leave the wound entirely open to the air and defy bacteriæ: etc. No kind of insect will touch the wound and it rapidly heals—often in serious cases, without a particle of suppuration.

All things considered, this is preferable to Lister's carbolic plan, for ordinary wounds at least.

**Diarrhœa of Children.**—Dr. Garrison, in the Lancet and Clinic, suggests the following:

| | | |
|---|---|---|
| ℞ | Carb. iron | gr. i. |
| | Bismuth subnit | gr. ij. |
| | Cerium oxalate | gr. i. |
| | Opii pulv | gr. 1-20. |

Mix for one dose to child six months old, and to be increased owing to age, every two to four hours.

**Milk Diet in Acute Rheumatism.**—M. Biot, in an important work published in the *Revue Mensuelle de Medecine et de Chirurgie*, exposes new ideas on the nature of acute rheumatism. The treatment must be started as soon as possible, and pure milk must be used at the exclusion of any other food. The quantity may vary according to taste and habits of the patient; the minimum dose being one quart a day, which can be increased to two, and even three quarts.

It is necessary that the milk be taken in small quantity and at regular intervals.

In a short time—from three to seven days—the patient becomes convalescent. When the pains have ceased and the fever has subsided, the treatment must be continued with mixed food, but alcohol, wine and meat must be dispensed with for some time, otherwise a relapse would surely be the result.

**Ergotin Hypodermics in Epistaxis.**—Dr. Porack cites three cases of obstinate nasal hemorrhage, each of which was promptly arrested by a single hypodermic of ergotin. His formula was:

| | | | |
|---|---|---|---|
| ℞ | Bonjean's ergotin | 2 grammes. | |
| | Glycerin | 30 " | M |

Twenty drops hypodermically in the lip or cheek.—*La Tribune Medicale.*

**To Test for Albumen.**—Da Costa says drop the fluid slowly down the side of the test-tube upon the nitric acid. If any albumen be present, an opaque white ring is seen to cover the surface of the acid. This is the most delicate test with which I am acquainted.—*Courier of Medicine.*

